# Test your knowledge and understanding

**Published:** 2012

This page is designed to test your understanding of the concepts covered in this issue and to give you an opportunity to reflect on what you have learnt. If anything has changed or improved as a result of reading this issue, tell us about it. Write to The Editor using the contact details provided on page 22. The multiple True/False questions were produced in collaboration with the International Council of Ophthalmology (ICO) and the Diagnose This Quiz is provided courtesy of the Ophthalmic News and Education (ONE®) Network of the American Academy of Ophthalmology.

**Table:** 

**1.**	**Think about welcoming patients into our eye service and improving patient flow. Which of these statements are true, and which are false?**	**True**	**False**
**a**	It is helpful to locate cashiers and drug dispensaries near the entrance of the hospital.	**□**	**□**
**b**	Before making changes in your eye clinic, it is important to find out what your staff think.	**□**	**□**
**c**	It is unlikely that you will be able to meet the needs of patients and the clinic team at the same time – usually you will have to choose one or the other.	**□**	**□**
**d**	Patients generally do not mind waiting a long time if they are able to come and go without losing their place in the queue.	**□**	**□**
**2.**	**Think about finding out what patients think and improving patients' experience**	**True**	**False**
**a**	Other health services, institutions, or organisations can help us to understand what patients think about our eye services.	**□**	**□**
**b**	Most patients value clinical outcomes more than they value being treated kindly.	**□**	**□**
**c**	Open-ended survey questions are easy and quick to analyse.	**□**	**□**
**d**	Patients responding to exit surveys are more likely to be honest if they are interviewed by someone who is not working in the eye clinic.	**□**	**□**
**3.**	**Think about taping an eyelid closed and tape correction for lower lid entropion**	True	False
**a**	When performing simple ophthalmic procedures, it is not always necessary to explain everything to the patient.	**□**	**□**
**b**	Tape correction for lower lid entropion can reduce the risk of exposure keratitis.	**□**	**□**
**c**	After taping an eyelid closed, always check that closure is effective.	**□**	**□**
**d**	Tape correction of the lower lid can provide temporary relief for patients suffering from trichiasis.	**□**	**□**
**4.**	**Think about checking and replacing fuses**	**True**	**False**
**a**	A visible gap in the wire or a metallic smear inside the glass indicates that the fuse must be replaced.	**□**	**□**
**b**	If a fuse blows immediately after you replace it, try at least twice more before callinga technician.	**□**	**□**
**c**	Always disconnect a device from the electrical system (generator, battery, or mains electricity) before replacing a fuse.	**□**	**□**
**d**	If a device is needed urgently, it is permissible to replace a fuse with foil or another object.	**□**	**□**	□

## ANSWERS

1. **a. False.** Where possible, charge a single fee for all services and place cashiers and dispensaries near the exit. This will prevent unnecessary back and forth movements. **B. true. 1. c. False.** See the examples in Table 1 on page 31. **d. true.**
2. **a. true. b. False.** If patients don't come to the clinic because they fear being badly treated, good clinical outcomes are of no use to them. **c. False.** Closed-ended questions are quick to analyses. Open-ended questions take longer. **d. true.** Patients may be reluctant to say something negative to staff working in the clinic as they may fear it will affect the care they receive.
3. **a. False.** Always explain to the patient what you are going to do. **b. False.** After taping the lower eyelid, check that the eye can still close, or else the patient may be at increased risk of exposure keratitis. **c. true.** Ask the patient to try to open both eyes. d. true.
4. **a. true.** Both indicate that the fuse has ‘blown’. **b. False.** A technician must service the equipment before it can be used again. **c. true. d. False.** Using foil or another object to replace a fuse is dangerous and can lead to electrocution, fires and damaged equipment.

## Dignose This Quiz

**Figure:**
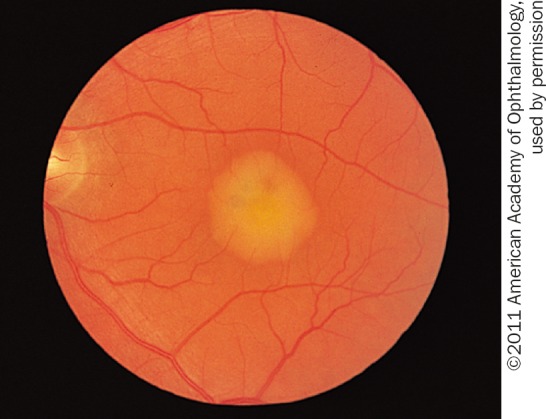


A12-year-old boy fails his school vision screening test. His medical history is benign. Middle-aged relatives of both sexes for three generations reportedly have had central visual loss. His best-corrected visual acuities are 20/80 OD and 20/20 OS. The disc, vessels, and retinal periphery of both eyes appear normal, but the macula of the right eye has a fibrotic scar and the left eye shows a yellow, round, circumscribed lesion of one disc diameter that blocks fluorescence on fluorescein angiogram. The ERG is normal, but the electro-oculogram ratio (light peaK/dark trough) is reduced. What is the most likely diagnosis?

- Neuronal ceroid lipofuscinosis
- Bardet-Biedl syndrome
- Best's vitel I form macular dystrophy
- Stargardt's disease

## ANSWERS

**The most likely diagnosis in this case is Best's disease.** Neuronal ceroid lipofuscinosis causes a hyperfluorescent bull's-eye maculopathy from generalised cone dystrophy, with seizures and progressive dementia in school-age years. In Stargardt's disease, the macular RPE atrophy causes hyperfluorescence against the abnormally dark background of the “silent choroid” sign. Bardet-Biedl syndrome frequently has atrophic maculopathy, but the diffuse photoreceptor loss causes reduced ERG amplitudes by a young age. Bardet-Biedl syndrome is autosomal recessive, and patients frequently have extra digits on their hands or feet. In Best's vitelliform macular dystrophy, visual acuity typically is excellent until the “egg yolk” ruptures or involutes. The yellow lesion is hypofluorescent in the early phases of the fluorescein angiogram but can stain and become hyperfluorescent in the later stages. The condition is transmitted from an affected parent as an autosomal dominant trait. The electro-oculogram is characteristically subnormal despite a normal ERG.

